# What Is the Tech Missing? Nutrition Reporting in Type 1 Diabetes

**DOI:** 10.3390/nu16111690

**Published:** 2024-05-29

**Authors:** Nicole Lubasinski, Hood Thabit, Paul W. Nutter, Simon Harper

**Affiliations:** 1 Department of Computer Science, The University of Manchester, Manchester M13 9PL, UK; p.nutter@manchester.ac.uk (P.W.N.); simon.harper@manchester.ac.uk (S.H.); 2 Diabetes, Endocrine & Metabolism Centre, Manchester Royal Infirmary, Manchester University NHS, Manchester M13 9WL, UK; hood.thabit@mft.nhs.uk; 3 Division of Diabetes, Endocrinology and Gastroenterology, School of Medical Science, The University of Manchester, Manchester M13 9PL, UK

**Keywords:** nutrition reporting, type 1 diabetes, T1D, mHealth, self-management, technology, apps, bolus targeting solution (BTS)

## Abstract

Introduction: Type 1 Diabetes (T1D) presents self-management challenges, requiring an additional 180 daily decisions to regulate blood glucose (BG) levels. Despite the potential, T1D-focused applications have a 43% attrition rate. This work delves into the willingness of people living with T1D (PwT1D) to use technology. Method: An online questionnaire investigated the current practices for carbohydrate estimation, nutritional tracking, and attitudes towards technology engagement, along with hypothetical scenarios and preferences regarding technology use. Results: Thirty-nine responses were collected from PwT1D (*n* = 33) and caregivers (*n* = 6). Nutrition reporting preferences varied, with 50% favoring ‘type and scroll’ while 30% preferred meal photographing. Concerning the timing of reporting, 33% reported before meals, 55% after, and 12% at a later time. Improved Time in Range (TIR) was a strong motivator for app use, with 78% expressing readiness to adjust insulin doses based on app suggestions for optimizing TIR. Meal descriptions varied; a single word was used in 42% of cases, 23% used a simple description (i.e., “Sunday dinner”), 30% included portion sizes, and 8% provided full recipes. Conclusion: PwT1D shows interest in using technology to reduce the diabetes burden when it leads to an improved TIR. For such technology to be ecologically valid, it needs to strike a balance between requiring minimal user input and providing significant data, such as meal tags, to ensure accurate blood glucose management without overwhelming users with reporting tasks.

## 1. Introduction

Type 1 Diabetes (T1D) is an autoimmune condition characterized by the inhibition of insulin production and the body’s inability to self-regulate blood glucose (BG) levels within optimal levels [1]. Managing T1D involves self-monitoring and self-management to maintain target BG levels, focusing on carbohydrate counting to determine the required exogenous insulin for post-meal BG control [1,2]. This need to self-manage requires people living with T1D (PwT1D) to make an additional 180 daily decisions to regulate their BG levels [3]. Therefore, diabetes-related technology and applications (apps) have emerged as valuable resources in alleviating this burden [4]. Carbohydrate counting, often deemed challenging, frequently leads to errors, even with commonly consumed foods [5]. Consequently, T1D management apps, or digital logbooks [6], predominantly focus on carbohydrate logs and diet recording to manage insulin dosing but often lack the adaptive feedback needed to offer accurate personalized decision support [7,8]. Despite the benefits for BG control demonstrated by the use of nutrition reporting platforms [9], challenges persist in estimating portion sizes and usability, particularly in non-English-speaking populations [10,11,12,13]. While the error rate in carbohydrate estimation varies based on individual skill, food complexity, and measurement accuracy, it is estimated to be 25%, which directly impacts BG control and ultimately the time spent within the target blood glucose range of 3.9–10 mmol/L (Time in Range (TIR)) [14,15,16,17,18]. Diabetes-targeted apps have been proven to enhance disease-specific knowledge and complication awareness by monitoring BG, insulin doses, and carbohydrate intake [19,20]. However, despite the reported benefits, challenges include reporting burdens and obstacles to sustained user engagement, with app usage declining once users reach their desired goals, such as an improved TIR [9,11]. Improved TIR goals are linked to a reduction in complication risk and improved patient outcomes, including lower HbA1c and improved quality of life [21,22].

This study seeks to proactively assess the level to which PwT1D are willing to engage with technology for an indefinite period. Further, it seeks to identify if a tool, offering accurate BG prediction and increased TIR, could motivate PwT1D to engage with technology and provide more detail.

## 2. Materials and Methods

The study employed a cross-sectional, single-center design with participants recruited through social media groups that specifically target PwT1D. In addition, PwT1D who were known to the research lab were contacted and asked to complete the online survey. Recruitment materials outlined the purpose of the study and directed potential participants to an online questionnaire hosted on the Qualtrics platform [23]. Participants were provided with a participant information sheet and provided electronic consent before proceeding with the questionnaire. The study was approved on 13 June 2022, by the University of Manchester, Department of Computer Science Institutional Ethics Committee (2022-14254-23883). Data collection occurred between August 2022 and November 2023.

The questionnaire developed for this research, consisted of three main sections. The first section (Q1–Q6) aimed to collect information on participants’ current practices related to carbohydrate estimation, nutritional tracking, and attitudes toward technology engagement. Participants were asked about their clinical diagnosis of T1D or their support for a family member with T1D. Multiple-choice questions were used to ascertain current practices regarding carbohydrate calculation tools, portion size determination, and tracking of nutrients other than carbohydrates. Additionally, a Likert scale [24]-type question assessed participants’ stress levels associated with carbohydrate counting. The second section (Q7–Q9) of the questionnaire explored hypothetical scenarios of recording nutritional intake at varying levels of nutrition reporting detail when different levels of TIR are promised. Using a standardized food example of ‘Hawaiian pizza’, participants were presented with multiple-choice questions accompanied by optional open-text responses to determine the level of detail they would be willing to use to report their nutritional intake to achieve an improved TIR.

The third section (Q10 and Q11) focused on participants’ preferences and experiences regarding the use of technology for tracking nutritional intake. Practical aspects of food reporting were assessed using multiple-choice questions concerning the method and timing of reporting. Participants were also asked to list, in natural detail, the foods they consumed most often (Q12). Demographic and baseline clinical information collected included the respondent’s current age, age at diagnosis of T1D (either for themselves or the person they care for), and, if available, the latest HbA1c result (Q13 and Q14). A charitable donation to Diabetes UK was made for each completed survey.

The specific questions posed to participants, along with response options, are provided in Appendix A.

## 3. Results

The study yielded 39 responses, of which 33 individuals had a T1D diagnosis and 6 cared for someone living with a T1D diagnosis. This includes children and adults who require support in managing their BG levels (Table 1).

**Figure 1:**
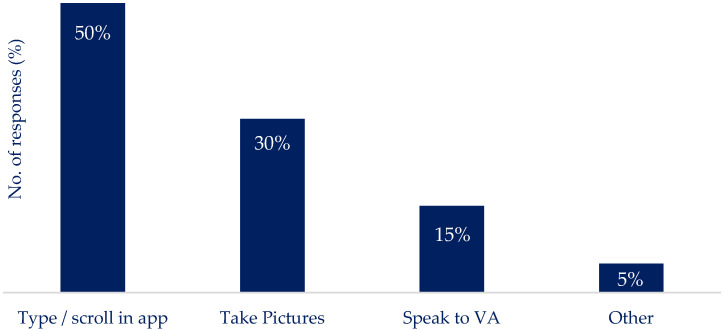
Preferred method of use to record food intake when using nutrition reporting apps: ‘type and scroll’ was the most used, followed by uploading photographs; speaking into a device was the least preferred method.

**Figure 2:**
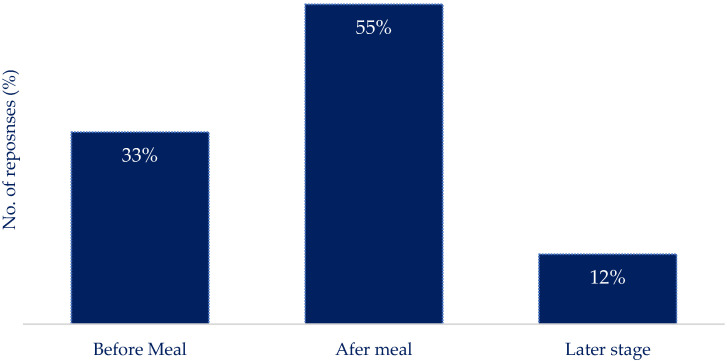
The reported habit of using nutrition reporting apps to record food intake. Reporting food intake after the event was considered the most practical.

This work considers nutrition reporting to be in the context of real-time decision-making to determine appropriate insulin dosing. The most practical method of nutrition reporting (Figure 1) was found to be ‘type and scroll’ type reporting (manually inputting foods eaten or searching for preloaded foods), i.e., selecting foods from a list, which was preferred by 50% of the respondents. The preference of the other methods included ‘taking and uploading photographs’ of a meal, preferred by 30%, and ‘speaking into a virtual assistant (VA) (e.g., Alexa)’ was preferred by 15%, while 5% indicated a preference to not report. Regarding the timing of reporting (Figure 2), 33% preferred reporting before consuming the meal, while 55% suggested it would be more effective to report after the meal, and 12% of participants indicated reporting their daily intake at a later stage (i.e., all at once at the end of the day).

**Figure 3:**
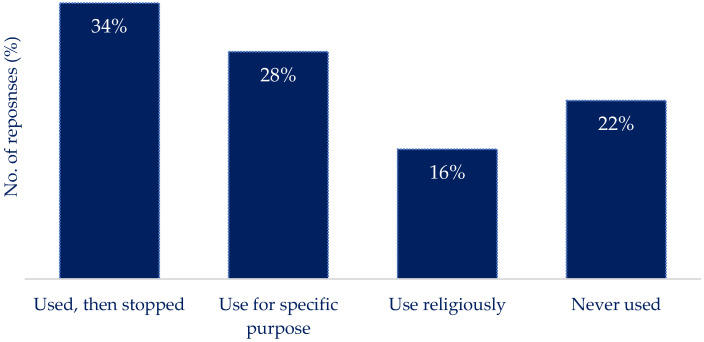
The reported engagement with nutrition reporting applications. There were varying levels of engagement with nutrition reporting applications among respondents. The largest segment, representing 34%, indicates individuals who have used these applications in the past but have since stopped.

**Figure 4:**
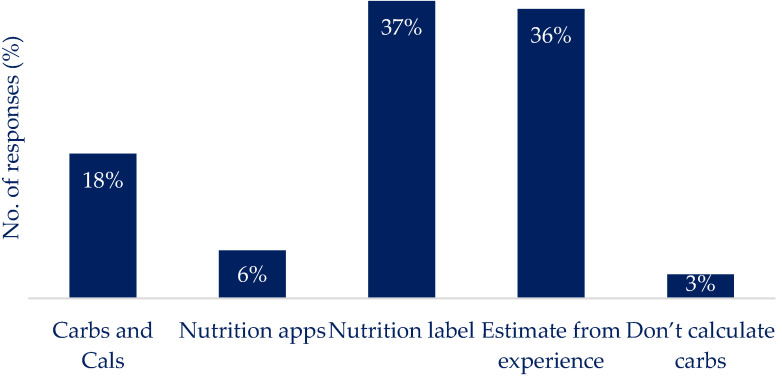
Distribution of information sources used to determine the carbohydrate content of food present in the meal. Reading off the nutrition label was the most common source of carbohydrate information, followed by estimations from previous experience. Less frequently used tools include ‘carbs & cals’ and nutrition reporting apps.

**Figure 5:**
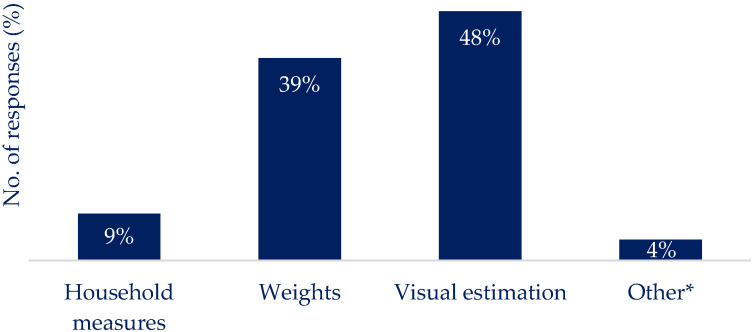
Preferred methods of determining portion size and carbohydrate content of the food consumed. Visual estimations were the most commonly used method, followed by weights and then household measures. Other* includes previous experience and specific quantities (i.e., 1 biscuit).

Figure 3 highlights the reported engagement with nutrition reporting apps: 16% reported regular use, and 34% reported using a nutrition reporting app and then stopping. The most common method for determining the carbohydrate content of a meal (Figure 4) was using the information on the nutrition label (37%), supported by using ‘estimation of the quantity present’ (48%) (Figure 5) to determine portion size. Previous experience was used to estimate the carbohydrate content (36%) (Figure 4), and the weight of the portion in conjunction with the carbohydrate content was used to determine the carbs per serving by 39% of respondents (Figure 5). Carbs and Cals [25] (a visual reference guide to determine carbohydrate content of foods) was used by 18% of respondents, and nutrition reporting apps (6%) were the least common source of information to determine carbohydrate content (Figure 4). The use of household measures to calculate carbohydrate content per portion was used by 9% of the respondents (Figure 5). Other methods of calculating carbs per portion relied on experience or specific quantities (e.g., one biscuit (Figure 5)).

**Figure 6:**
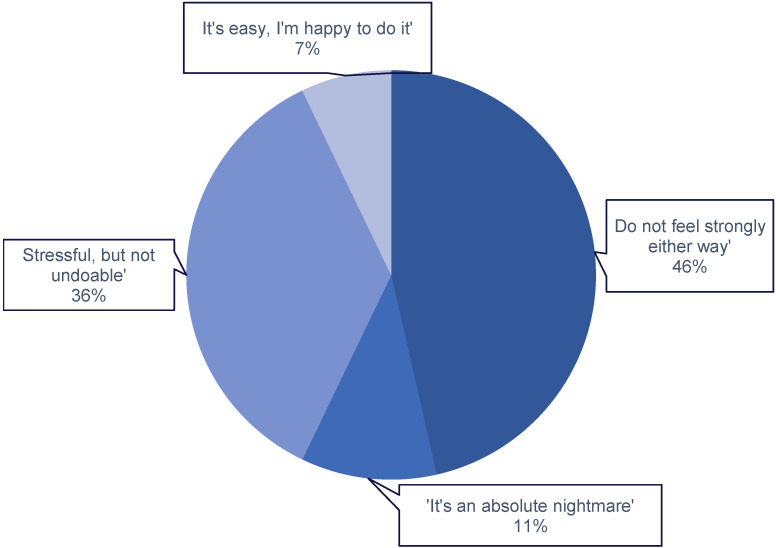
Overall perceptions of food reporting for people living with T1D. Ranging from ‘Easy, happy to do it’ through to an ‘An absolute nightmare’.

In terms of the burden of nutrition reporting (Figure 6), 36% considered it ‘stressful, but manageable’, 11% found it an ‘absolute nightmare’, 7% considered it ‘easy’, and 46% ‘did not feel strongly either way’ about carbohydrate counting. Participants were asked to describe commonly consumed foods in their natural level of detail (this could be as simple as ‘cheese sandwich’ or as detailed as ‘sandwich with whole meal bread, Emmental cheese, pickle, and mayonnaise’). A single word to describe the food consumed was used in 42% of cases, while a ‘simple description’ (i.e., Sunday dinner) was used in 23% of cases. Quantifiers, either number of portions or portion weight, were used in 30% of the meals reported. Other reporting styles, used by 8%, included a detailed description of the meal (i.e., “Wholegrain burrito with ground beef, tomato, bell pepper, black beans. Toppings: guacamole and creme fraiche”).

**Figure 7:**
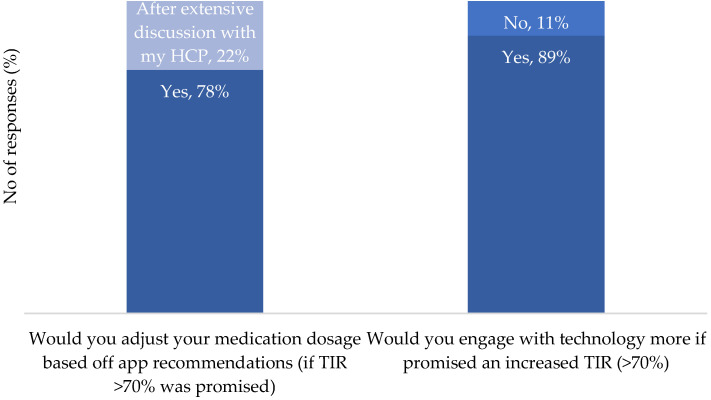
Motivation for engagement with technology: 78% of respondents would adjust medication dosages on a recommendation from an app if a >70% TIR was promised, while 22% would consult with a medical team before following the app recommendations. In terms of increasing the detail of reporting, 89% would engage with technology more and provide more detail to achieve an increased TIR.

It was seen that an app offering >70% TIR was appealing and a motivation for use for 89% of the respondents (Figure 7).

**Figure 8:**
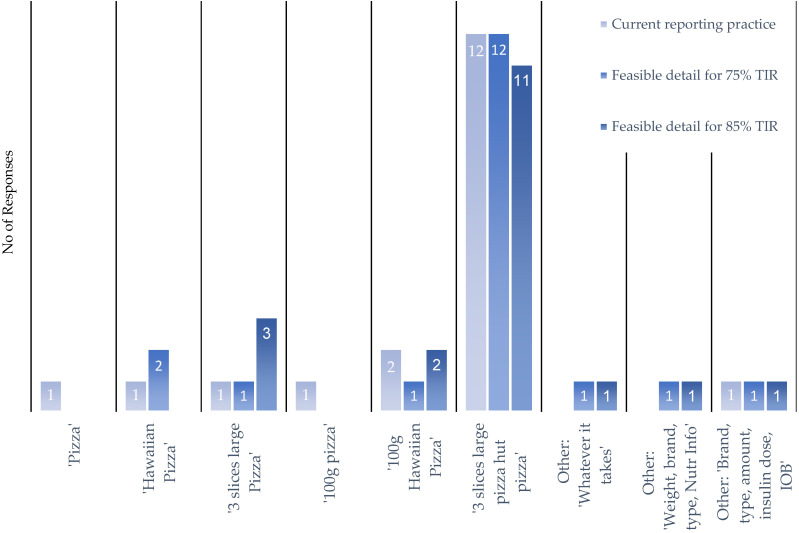
Level of detail in nutrition reporting considered feasible with increasing accuracy of prediction and improved TIR. Ranging from a single word or a more detailed recording (“pizza” or “Hawaiian pizza”), including quantity in either grams or servings (“100 g” or “3 slices”), or including quantity, brand, and food type (“3 slices of large (individual/12”) pizza from Pizza Hut”).

When questioned about the level of reporting detail deemed acceptable to achieve TIR, it was seen to not change with an increased TIR (Figure 8). In addition, should the app suggest medication adjustments to optimize TIR, 78% of respondents indicated they would modify insulin doses to optimize TIR, while 22% would consider adjusting the medication dose after considerable consultation with their medical team.

## 4. Discussion

The analysis of the results obtained from the study outlines perceptions and preferences for methods surrounding nutrition reporting among PwT1Ds and their caregivers. The results of this work reveal there is a range of preferred methods of reporting and tools used to determine the carbohydrate content and size of the meal. This holds implications for the design and implementation of nutrition reporting tools and technologies. For an app to encourage long term engagement, personalized reporting methods should be considered.

In terms of the timing of reporting (Figure 2), a majority of participants (55%) suggested that reporting after the meal would be preferential compared to reporting before consuming the meal or reporting daily intake at a later stage. This preference for post-meal reporting could be due to the convenience and accuracy of reporting actual consumption rather than planned intake. However, in. the context of using nutrition reporting for insulin dose decision making, reporting intake after the event would be problematic.

The level of detail in nutrition reporting varied among respondents, with a majority opting for simple descriptors to describe the food consumed. Current reporting practices of reporting the quantity and brand of food consumed are shown to be the ceiling for the detail of reporting deemed acceptable, regardless of the success offered by the app. Overall, the findings underscore the importance of understanding user preferences, behaviors, and challenges in nutrition reporting among individuals with T1D and their caregivers. These insights can inform the development of tailored interventions and support tools to enhance the management of T1D and improve the user experience in nutrition reporting.

Diabetes apps are typically assessed across six categories: self-monitoring, education, alerts, reminders, social media integration, and personal health record synchronization. However, due to variations in personalities and preferences, achieving a universally effective solution for app requirements is challenging [26]. Despite the identified optimal features for diabetes self-management apps, many available apps fail to incorporate them adequately, leading to poor long-term engagement [26]. Notably, free apps, which are often utilized by individuals with low health literacy and socioeconomic status, tend to lack features that enhance usability and understanding [27]. Even with this gap in T1D-focused apps, the work presented here indicates that PwT1D are motivated to engage with technology, showing that in developing ecologically viable technology, consideration of human interaction to meet users’ needs is needed [28]. Integrated mobile apps have shown reductions in HbA1c, but usability issues and time demands often lead to decreased user retention [29].

Quantifying engagement with apps poses challenges, with metrics like opening the app and time spent inside being used [30]. When investigating mHealth interventions, 80% of research participants engage with the minimal standard (logging in < twice), with a small proportion consistently using the app [8]. Attrition rates in short-term study periods exceed 30% for nutrition-focused apps and 20% for T1D self-management apps [8,13], indicating a significant challenge in retaining users in real-world settings [28]. This supports the findings presented here, with only 16% of respondents reporting to use nutrition reporting apps religiously, vs. 34% using an app for a period of time before stopping and 28% using an app for specific functions (i.e., changing diet or body shape). Clinician referrals for apps have shown increased engagement, but issues persist with inaccurate reporting, particularly in those with poorly controlled BG values [11,31].

When viewing T1D as a data-driven disease, the psychological burden is often overlooked, and due to this burden, patients often do not document self-management aspects of the treatment plan [32]. Engagement with general nutrition reporting apps relies heavily on the time and effort required for data input [33]. Justifications for low engagement, even in a motivated person, include that the positive aspects of the interface do not generate enough enthusiasm for active and frequent usage. It has also been noted that standard data visualization often creates confusion and increases cognitive overload [11]. To enhance user retention and cater to a wider audience, self-management tools offering varying levels of engagement, including food identification from images, have been proposed. Despite advancements in self-management tools, challenges remain in balancing simplicity and accuracy, with gamification strategies potentially yielding counterproductive effects [11,34].

The results from this work support the idea that carbohydrate content remains the primary focus of nutrition reporting among PwT1D, given its direct impact on BG variation [1,35]. This focus results in the frequent inclusion of food diaries and carbohydrate tracking features found in T1D-focused apps [19,36]. While weighted food diaries are considered the ‘gold-standard’ [37], digital diaries offer various input methods [38], with ‘type and scroll’ being preferred by the respondents of this study. Image-based methods of nutrition reporting, which were shown as a popular method of reporting in this work, offer advantages but struggle with food isolation and portion size estimation accuracy, especially with complex meals. It has been shown that carbohydrate estimation between image-based and traditional methods differs by 8 grams per day [39]. The variety of preferences in reporting methods shown here is supported in part by other research, which shows the choice of how to report nutritional information varies depending on demographic characteristics [40] and the input features offered by the app of choice [41]. Portion size estimation has emerged as a prominent source of error in both electronic and manual food diaries. Using visual estimation, as shown to be the preferred method of determining portion size in this work, is prominent to human error and reliant on the dish on which the meal is served in complex meals [42,43]. There are limitations associated with how foods are uploaded into apps, typically involving manual text searches or barcode scanning [5] and the natural and technological limitations of image-based methods [44].

The accuracy of nutrition reporting is based in part on memory, suggesting the method used and time between consuming food and reporting are important [45]. In PwT1D, using food reporting to determine the correct dose of bolus insulin needed to manage the glycemic response to the meal and compiling all the nutritional information toward the end of the day or at a later time may potentially influence TIR. This work shows that Pw T1D would prefer to report after the meal was consumed; this may be after consumption (55%), or at the end of the day (12%), which could affect the efficacy of the app if being used for T1D self-management, having an impact on postprandial glycemic control and decreasing TIR. In the context of nutrition reporting applications, obstacles to sustained utilization encompass the manual initiation of data recording, indicating that the mode of data collection may influence the motivation for prolonged engagement [46]. Notably, PwT1D tend to discontinue app usage upon achieving BG stability, diminishing the longevity of their involvement [11]. The impediments to sustained long-term usage can be systematically classified into several domains: individual factors (such as goals, settings, goal striving, motivation, routines, and lack of awareness or knowledge), technological aspects (including app features, usability, trustworthiness, technical issues, and financial costs), (non)-intended outcomes, and the social environment [46].

The results of this work suggest that offering a solution that requires a minimum amount of information from the user while providing considerable data (such as a meal tag that represents the meal as a whole) would allow for accurate BG management while limiting the burden of reporting. While additional work is needed to ascertain the exact features needed to retain users in the long term, offering an improved TIR is suggested as a motivator for increased engagement.

Using mobile nutrition reporting applications allows for food intake to be recorded in real time with remote access by HCPs to online food diaries, push reminder notifications, and the habitual nature of carrying a mobile phone around to encourage the continuous monitoring of intake [47]. This work supports the notion that the level of engagement with nutrition reporting platforms is motivated by the success of the outcome (i.e., increased TIR). However, obstacles to sustained utilization of nutrition reporting apps include manual data initiation and discontinuation upon achieving BG stability [11,46]. These obstacles can be categorized into individual factors, technological aspects, (non-)intended outcomes, and the social environment [46].

### Limitations and Future Work

One limitation of this study is the low number of responses received, which may affect the generalizability of the findings. Other limitations of this study include the nature of the data collection (an online questionnaire), meaning one cannot fully describe the population and may be subject to bias [48]. There was no exclusion of individuals with poor literacy skills, although the online format may have attracted a more literate demographic. Despite this, participants unfamiliar with concepts like ‘Hawaiian pizza’ were not excluded, though data analysis was limited to completed surveys, implying respondents were likely familiar with the examples provided. The methodology lends itself to a more technologically skilled individual, as increased use of technology has been linked to improved glycemic control in PwT1D [49]. Having this skill bias ingrained in the methodology results in limitations in extrapolating the results to the entire T1D population. To build on these findings, an in-person interview may allow for a more in-depth understanding of the motivations and barriers to engaging with technology for nutrition reporting purposes.

Future work should assess the integration of personalized features, such as real-time health insights and adaptive notifications, to better motivate users to increase app engagement for T1D management. Understanding and addressing the factors contributing to dropout rates, such as usability challenges, a lack of sustained motivation, and missing features or actionable advice, will be crucial for developing more effective and user-friendly diabetes management apps. The ultimate vision is to develop an ecologically viable BG prediction model that integrates seamlessly into the daily lives of people living with T1D, empowering them with precision and convenience in disease management.

## 5. Conclusions

In conclusion, this research investigates the receptiveness of PwT1D towards self-management applications focusing on nutritional tracking. It underscores the potential of advancing technology to alleviate the challenges of T1D management but highlights a gap between identified effective features and available applications. The study reveals a strong willingness among T1D patients to adopt technology, particularly for tracking nutrition, driven by the promise of improved TIR. This enthusiasm underscores the importance of developing technology that seamlessly integrates into daily life. Moving forward, this research provides valuable insights to inform the development of ecologically viable tools aimed at enhancing the T1D management experience, envisioning a future marked by personalized, precise, and convenient care.

## Figures and Tables

**Table 1:** Demographic characteristics and clinical profile of the study population.

	No. of Responses
Diagnosis of Type 1 Diabetes:	
Self	33
I support a family member with T1D	6
Duration of diabetes:	
0–5 years	5
5–10 years	0
10–15 years	0
15+ years	28
Not reported	6
Average HbA1c	6.8 ± 1.6%
Age of PwT1D at the time of the questionnaire:	
0–9	3
10–14	0
15–24	0
25–34	7
35–44	6
45+	18
Not reported	5

## Data Availability

Data is contained within the article.

## Appendix A

**Table A1:** Questions and answering options presented to participants via the Qualtrics Platform.

Q1: Have you been diagnosed with Type 1 Diabetes? Yes, I have a clinical diagnosis of Type 1 Diabetes No, but I support a family member with a clinical diagnosis of Type 1 Diabetes No, neither myself nor a family member has a clinical diagnosis of Type 1 Diabetes
Q2: How do you currently work out the amount of carbohydrates you are eating in a meal? (select all that apply) Carbs and Cal (book or app) Nutrition reporting app, i.e., MyFitnessPal (please indicate the app used) Reading off the nutrition label on food packaging Rough estimation based on previous experience I do not use carbohydrate content in my daily blood glucose management
Q3: When thinking about calculating the carbohydrates present in a meal, how do you measure your food? (select all that apply) Household measures (i.e., tablespoons, cups, etc.) Either weigh on a scale or weight taken directly from packaging Visual Estimations Other, please specify:
Q4: If you record your food intake, how do you feel about it? (Likert Scale) 1 = Do not feel strongly either way 3 = It’s easy, I am happy to do it 5 = Stressful, but not undoable 7 = It’s an absolute nightmare
Q5: Do you track/report any other nutrition information (i.e., total energy or vitamins and minerals)? Yes, I track everything No, these do not impact blood glucose levels Sometimes, when trying something new or trying to change body shape This is not something I have considered doing
Q6: If you think about engaging with technology around your nutritional intake, what would be the most practical way for you to do this? Type/Scroll and select from the food items in a smartphone app Take pictures of the meals and upload to a smartphone app Speak into a device (i.e., Siri or Alexa) Other:
Q7: Following on from the above scenario, to what level of detail would you record your nutritional intake if you were to report it by typing/scrolling or using a voice- activated interface (let us use a Hawaiian pizza as an example)? You can choose from the below options: Record it as “Pizza” Record it as “Hawaiian pizza” Record it as “3 slices of large (individual/12”) pizza” Record it with the brand or restaurant included, i.e., “3 slices of large (individual/12”) pizza from Pizza Hut” 100 g of “Pizza” 100 g of “Hawaiian Pizza” Other, please specify:
Q8: If providing more detail of the food you eat would allow an application to offer improved blood glucose control. Ensuring an HbA1C of 7% and at least 75% time in blood glucose target range. To what level of detail would you be willing to record your nutritional intake. Record it as “Pizza” Record it as “Hawaiian pizza” Record it as “3 slices of large (individual/12”) pizza” Record it with the brand or restaurant included, i.e., “3 slices of large (individual/12”) pizza from Pizza Hut” 100 g of “Pizza” 100 g of “Hawaiian Pizza” Other, please specify:
Q9: If providing more detail of the food you eat would allow an application to offer improved blood glucose control. Ensuring an HbA1C of below 6.5% and at least 80% time in blood glucose target range. To what level of detail would you be willing to record your nutritional intake. Record it as “Pizza” Record it as “Hawaiian pizza” Record it as “3 slices of large (individual/12”) pizza” Record it with the brand or restaurant included, i.e., “3 slices of large (individual/12”) pizza from Pizza Hut” 100 g of “Pizza” 100 g of “Hawaiian Pizza” Other, please specify:
Q10: If you were to make use of an application to report your nutritional intake, what would be the most effective way to report the information? Input the information BEFORE a meal or snack Input the information AFTER a meal or snack Input all the information for the day at a later stage (i.e., at the end of the day or the next morning)
Q11: If you currently use/have used technology to track nutritional intake. What was your experience? Use it to track more than 80% of meals and snacks Used it for a few weeks, then lost interest and stopped Used it often for a while, now use it from time to time to get a better understanding of what is eaten in a day or if patterns change and re-evaluation is needed I have never used technology to track my nutritional intake
Q12: Thinking about the foods you eat most often (no need to fill in all the spaces). List them below in the amount of detail you would naturally use. For example, this can be as straightforward as a sandwich or as detailed as ham and cheese sandwich on whole wheat with margarine and mayo or anything in between.
Q13: Are you willing to share your HbA1c results with us and your current treatment plan? If yes, please answer the below: Latest HbA1c result Carb-to-Insulin Ratio Total Units of Insulin Daily Insulin used No, I do not wish to share this information
Q14: How old were you, or your family member, when diagnosed with Type 1 Diabetes? 0–4 years of age 5–10 years of age 11–15 years of age 16–20 years of age 20+ years of age
